# Knowledge mapping of mitochondrial calcium uniporter from 2011 to 2022: A bibliometric analysis

**DOI:** 10.3389/fphys.2023.1107328

**Published:** 2023-01-20

**Authors:** Deng Pan, Lin Xu, Dazhuo Shi, Ming Guo

**Affiliations:** ^1^ Xiyuan Hospital of China Academy of Chinese Medical Sciences, Beijing, China; ^2^ National Clinical Research Centre for Chinese Medicine Cardiology, Xiyuan Hospital of China Academy of Chinese Medical Sciences, Beijing, China; ^3^ Graduate School of Beijing University of Chinese Medicine, Beijing, China; ^4^ Gynecological Department of Traditional Chinese Medicine, China-Japan Friendship Hospital, Beijing, China

**Keywords:** mitochondrial calcium uniporter, calcium homeostasis, citespace, VOSviewer, bibliometric

## Abstract

**Background:** Calcium uptake research has a long history. However, the mitochondrial calcium uniporter (MCU) protein was first discovered in 2011. As investigations of mitochondrial calcium uniporter represent a new research hotspot, a comprehensive and objective perspective of the field is lacking. Hence, this bibliometric analysis aimed to provide the current study status and trends related to mitochondrial calcium uniporter research in the past decade.

**Methods:** Articles were acquired from the Web of Science Core Collection database. We quantified and visualized information regarding annual publications, journals, cocited journals, countries/regions, institutions, authors, and cocited authors by using CiteSpace 5.8. R3 and VOSviewer. In addition, we analysed the citation and keyword bursts related to mitochondrial calcium uniporter studies.

**Results:** From 2011 to 2022, 1,030 articles were published by 5,050 authors from 1,145 affiliations and 62 countries or regions. The country with the most published articles was the United States. The institution with the most published articles was the University of Padua. Rosario Rizzuto published the most articles and was also the most cocited author. Cell Calcium published the largest number of articles, whereas *Journal of Biological Chemistry* had the most cocitations. The top 5 keywords related to pathological processes were oxidative stress, cell death, permeability transition, apoptosis, and metabolism. MICU1, calcium, ryanodine receptor, ATP synthase and cyclophilin D were the top 5 keywords related to molecules.

**Conclusion:** mitochondrial calcium uniporter research has grown stably over the last decade. Current studies focus on the structure of the mitochondrial calcium uniporter complex and its regulatory effect on mitochondrial calcium homeostasis. In addition, the potential role of mitochondrial calcium uniporter in different diseases has been explored. Current studies mostly involve investigations of cancer and neurodegenerative diseases. Our analysis provides guidance and new insights into further mitochondrial calcium uniporter research.

## 1 Introduction

Mitochondria are key organelles involved in the regulation of energy production, and calcium homeostasis plays a key role in the maintenance of mitochondrial structure and function (Zhang et al., 2022a). Studies on mitochondrial calcium regulation began 60 years ago. Deluca and his colleague first found that mitochondria take up a large amount of calcium when cellular energy is required (Deluca and Engstrom, 1961). Hence, the concept of mitochondrial calcium uptake, termed MCU, was established and has been studied since the 1960s. After ruthenium 360 (Ru360) (a specific mitochondrial calcium uptake inhibitor) was found, various calcium uptake and efflux pathways were defined (Rizzuto et al., 1987). In 1979, Harworth and Hunter discovered that there is a maximum mitochondrial calcium content (called Calcium capacity retention). Once the content of calcium exceeds the mitochondrial capacity, the permeability of the mitochondrial inner membrane increases, and molecules of under 1 kD (molecules of under 1.5 kD are permeable) pass through a pore called the mitochondrial permeability transition pore (mPTP) (Haworth and Hunter, 1979). Studies were conducted steadily until 2011. Protein CCD109A was found to be responsible for mitochondrial calcium uptake, and the MCU acronym was subsequently used to represent the mitochondrial calcium uniporter protein (abbreviated MCU hereafter) (Baughman et al., 2011; De Stefani et al., 2011). Soon after the discovery of MCU, it was found that MCU is a part of a complex called the MCU complex (Baradaran et al., 2018). The MCU complex is involved in various diseases and pathology processes, such as Alzheimer’s disease (Esteras and Abramov, 2020), ischaemic neurodegeneration (Medvedeva and Weiss, 2014), ischaemia‒reperfusion injury (Li et al., 2021a), pancreatic cancer (Wang et al., 2022b), liver steatosis (Zhang et al., 2022c), and Barth syndrome (Ghosh et al., 2020). Thus, studies targeting the MCU complex and its components are attracting increasing attention, which sheds light on mitochondrial calcium homeostasis and related diseases.

The MCU complex comprises MCU, MICU1, MICU2, MICU3, and EMRE (essential MCU regulator) (Nguyen et al., 2018). MCU was the first component discovered in the MCU complex. De Stefani et al. first discovered the MCU protein and found that mitochondrial calcium uptake was significantly inhibited in MCU-silenced HeLa cells (De Stefani et al., 2011). MCU knockout (KO) prevents mitochondrial swelling and dysfunction under high calcium levels, which is associated with the sensitivity of the mitochondrial permeability transition pore (mPTP) (Pan et al., 2013). Mitochondrial calcium uniporter dominant negative subunit b (MCUb) is a homologue of MCU, which shares 50% of the sequence of MCU (Raffaello et al., 2013). When MCUb replaces one MCU in the MCU tetramer, the dominant negative effect of MCUb leads to inhibition of calcium uptake, which is a notable mechanism in maintaining mitochondrial calcium homeostasis. MICU1, MICU2, and MICU3 are three EF-hand proteins that regulate the activity of MCU (Plovanich et al., 2013; Patron et al., 2019). Among the three regulators, only MICU1 can bind with MCU directly (Kamer et al., 2017). MICU1 inhibits calcium uptake under low calcium levels, whereas calcium uptake is enhanced under elevated calcium levels (Csordás et al., 2013). MICU2 and MICU3 cannot bind to MCU directly. Instead, they regulate calcium uptake by binding to MICU1. MICU2 is thought to have the opposite effect as MICU1 in regulating mitochondrial calcium, which reduces the activity of MCU (Patron et al., 2019). MICU3 enhances mitochondrial calcium uptake by binding to MICU1, which is highly expressed in neuronal tissue (Patron et al., 2019). EMRE is a 10 kD protein with a single transmembrane domain that is expressed from the SDMT1 gene and plays an essential role in mitochondrial calcium uptake in metazoans (Sancak et al., 2013). EMRE binds to MCU to form a minimal functional unit of the MCU complex (Wang et al., 2019).

Despite MCU being a recently found molecule, the number of studies on MCU and the MCU complex is growing rapidly, which suggests that it is a promising target in disease or pathology processes related to mitochondria and calcium homeostasis. In addition, as a rapidly developing field, reviews on MCU have been published, which mainly focused on calcium signaling, calcium homeostasis and relative diseases (De Stefani et al., 2016; Raffaello et al., 2016; Wang and Wei, 2017; Marchi et al., 2020; Woods and Wilson, 2020; Murphy and Steenbergen, 2021; Garbincius and Elrod, 2022). However, to the best of our knowledge, there has been no bibliometric study on the field of MCU. Bibliometric analyses focus on various information from published articles, such as authors, countries, citations, keywords, etc., to provide an evidence map of an existing research category and present a reference for subsequent researchers.

Here, we conducted a bibliometric analysis on MCU by using two bibliometric analysis software programs, CiteSpace and VOSviewer, to quantify and visualize the information from published articles. In addition, we explored the development trends in MCU research over the past 10 years and identified the hotspots in this field.

## 2 Materials and methods

### 2.1 Data collection

Our bibliometric analysis data were derived from the Web of Science Core Collection (WoSCC), which includes Science Citation Index Expanded (SCIE), Social Science Citation Index (SSCI), and Emerging Sources Citation Index (ESCI); the WoSCC is widely used in bibliometric analyses (Shi et al., 2022). Our search was conducted from 1 January 2011 to 23 June 2022. The search terms were set as follows: [TS = (“mitochondrial calcium uniporter” OR “mitochondrial calcium uniporter complex”)] AND [Publication type = (Article)] AND [Language = (English)]. We exported our file as a “plain text file,” and the record content was set as “full record and cited references.” For further analysis, the files were renamed “download_.txt,” which is readable in CiteSpace.

### 2.2 Data analysis

In our bibliometric analysis and visualization, we used CiteSpace 5.8. R3 (Chaomei Chen, 2006), VOSviewer 1.6.18 (Nees Jan van Eck and Ludo Waltman, 2010), and Microsoft Excel 2013. CiteSpace is a tool for bibliometric analysis and visualization that can detect the dynamics of a specific scientific field by analysing authorship, institution, keywords, and citing information. Moreover, CiteSpace is able to find keywords and citation bursts. The settings used for the CiteSpace analysis were as follows: time span (2011–2022), years per slice 1), selection criteria (Top *N* = 100), and minimum duration of burst (2 years). All other settings were the default values. VOSviewer is a tool for visualizing bibliometric analysis results. In a VOSviewer cluster map, the same colour belongs to the same cluster. In a density map, a larger word size and higher opacity of yellow indicate a higher density. Regarding annual publication analysis, we used Microsoft Excel. All impact factors (IFs) and H-indexes of the scholars were obtained from Web of Science on 23 June 2022.

## 3 Results

### 3.1 Publication trends and analysis of countries/regions and affiliations

**FIGURE 1:**
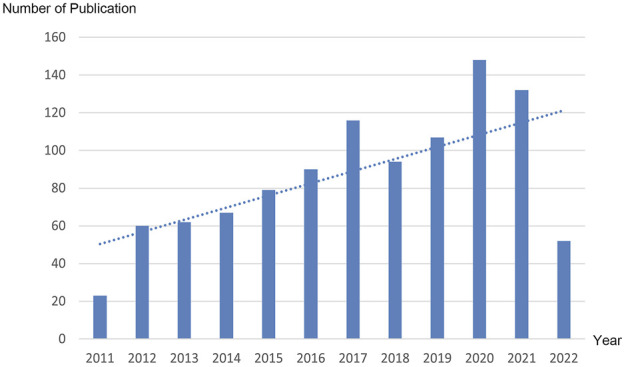
Trend of the number of publications related to MCU annually from 2011 to 2022. Dotted line shows the linear prediction of the number of publications.

**FIGURE 2:**
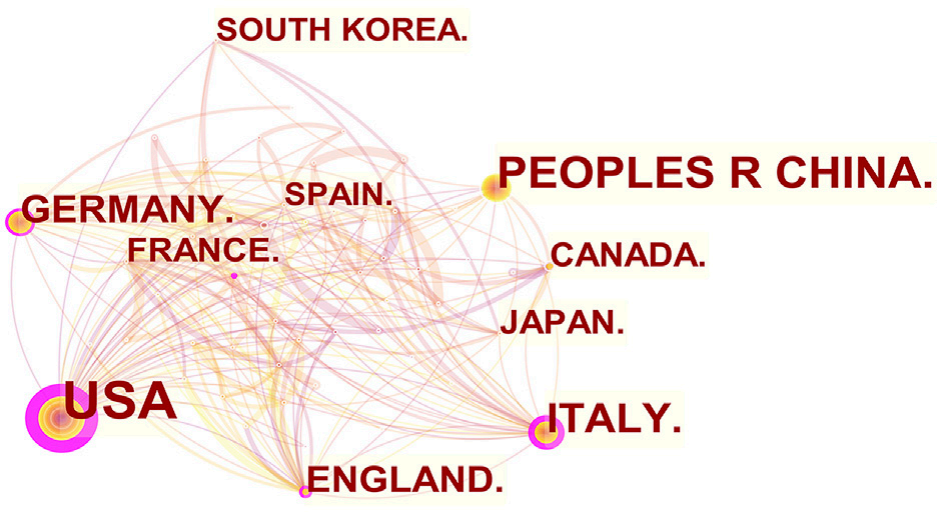
The co-occurrence map of countries/regions in MCU studies (*n* ≥100). The node size reflects the co-occurrence frequencies, and the links indicate the co-occurrence relationships. Colors vary from purple to red as time goes from 2011 to 2022, and nodes with purple round means high centrality (≥0.1).

From 2011 to 2022, there were a total of 1,030 publications from 62 countries and 1,145 affiliations. The number of MCU-related articles increased as a whole throughout the last decade, and it can be expected that the increasing trend will continue (Figure 1). The United States published the most articles (*n* = 446), and China (*n* = 193) and Italy (*n* = 127) published the second and third most articles. Regarding centrality, the United States occupied first place in the world (centrality = 0.71), followed by Italy (centrality = 0.20) and England (centrality = 0.18) (Figure 2). The results suggested that the United States is the main node of studies concerning MCU. In contrast, the centrality of China was 0.02, suggesting that China is not a key node in related studies despite the large number of publications. In terms of affiliations, the University of Padua published the most articles (*n* = 90), followed by Thomas Jefferson University (*n* = 37) and Temple University (*n* = 33). The University of Padua (centrality = 0.20) and Thomas Jefferson University (centrality = 0.17) had high centrality results (Table 1).

**TABLE 1:** Top 10 institutions published articles related to MCU.

Rank	Institution	Count	Centrality
1	University of Padua	90	0.2
2	Thomas Jefferson University	37	0.17
3	Temple University	33	0.05
4	National Research Council Institute of Genetic and Biomedical Research–Unità Operativa di Supporto Milan	30	0.07
5	Massachusetts General Hospital	27	0.06
6	Harvard Medical School	20	0.03
7	University of Ferrara	19	0.06
8	Ben-Gurion University of the Negev	18	0.05
9	University of Pennsylvania	16	0.01
10	Broad Institute	15	0.01

### 3.2 Analysis of authors and coauthors

A total of 5,050 authors were involved in MCU studies. Among them, 37 authors published 10 articles or more. R. Rizzuto published the most related articles (*n* = 40), followed by M. Madesh (*n* = 28) and Vamsi K. Mootha (*n* = 23) (Table 2).

**TABLE 2:** Top 10 authors and co-cited authors related to MCU.

Rank	Author	Count	H Index	Co-cited author	Count	H Index
1	Rosario Rizzuto	40	105	Diego De Stefani	771	33
2	Muniswamy Madesh	28	57	Rosario Rizzuto	613	105
3	Vamsi K Mootha	23	82	Joshua M Baughman	582	10
4	Anna Raffaello	17	23	Gyorgy Csordas	511	39
5	Santhanam Shanmughapriya	17	31	Karthik Mallilankaraman	489	27
6	Paolo Pinton	17	89	Kimberli J Kamer	363	22
7	Diego De Stefani	16	33	Fabiana Perocchi	350	22
8	Ildiko Szabo	15	60	Yuriy Kirichok	347	20
9	Cristina Mammucari	12	31	Maria Patron	332	10
10	Elizabeth Murphy	12	79	Anna Raffaello	291	23

**FIGURE 3:**
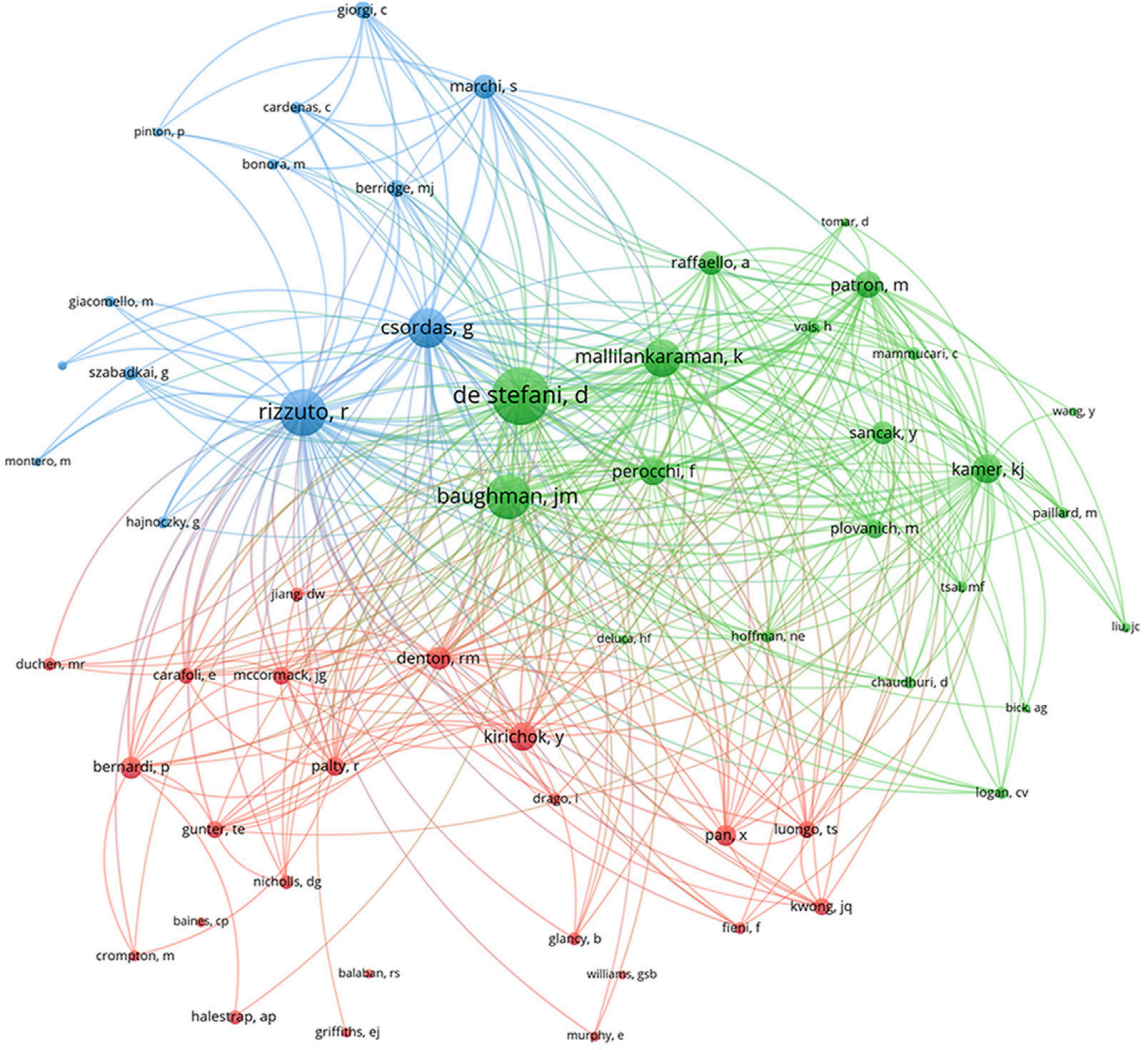
Cocited authors with over 100 citations by cluster map. The same colour indicate the same cluster. The larger the circle, the more the authors were cited.

Cocited authors are the authors who were cited in one article. Among 28,205 cocited authors, 57 authors had over 100 cocitations. Figure 3; Table 3 shows the authors with over 100 citations by cluster mapping and the name list of authors in three clusters. The same colour indicates the same cluster, and the larger the circle, the more the authors were cited. According to the cluster map, there were three colours in Figure 3, representing three clusters of authors. De Stefani D, Rizzuto R, Baughman JM and Csordas G were the four most cocited authors. There were intense links within and between clusters, and notably, the four most cocited authors also linked with each other as the centrality in MCU studies. The top 10 cocited authors and their counts of citations are presented in Table 2.

**TABLE 3:** Clusters of authors with over 100 cocitations.

Cluster 1	Cluster 2	Cluster 3
Baines CP	Baughman JM	Berridge MJ
Balaban RS	Bick AG	Bonora M
Bernardi P	Chaudhuri D	Cardenas C
Carafoli E	De Stefani D	Csordas G
Crompton M	Deluca HF	de brito OM
Denton RM	Hoffman NE	Giacomello M
Drago I	Kamer KJ	Giorgi C
Duchen MR	Liu JC	Hajnoczky G
Fieni F	Logan CV	Marchi S
Glancy B	Mallilankaraman K	Montero M
Griffiths EJ	Mammucari C	Pinton P
Gunter TE	Paillard M	Rizzuto R
Halestrap AP	Patron M	Szabadkai G
Jiang DW	Perocchi F	
Kirichok Y	Plovanich M	
Kwong JQ	Raffaello A	
Luongo TS	Sancak Y	
McCormack JG	Tomar D	
Murphy E	Tsai MF	
Nicholls DG	Vais H	
Palty R	Wang Y	
Pan X		
Williams GS		

### 3.3 Analysis of journals and cocited journals

In total, 400 journals have published articles related to MCU, of which 49 have published more than five articles. Among them, Cell Calcium accounted for the most publications (*n* = 46), followed by *Journal of Biological Chemistry* (*n* = 36) and Cell Reports (*n* = 26) (Table 4).

**TABLE 4:** Top 10 journals with most publications, citations and co-citations related to MCU.

Rank	Journal	Publications	Impact factor	Cited journal	Count	Impact factor	Co-cited journal	Count	Impact factor
1	Cell Calcium	46	6.817	Nature	4,329	49.962	Journal of Biological Chemistry	4,163	5.517
2	Journal of Biological Chemistry	36	5.517	Proceedings of the National Academy of Sciences of the United States of America	1807	11.205	Nature	3,984	49.962
3	Cell Reports	26	9.423	Cell Reports	1,508	9.423	Proceedings of the National Academy of Sciences of the United States of America	3,025	11.205
4	Journal of Molecular and Cellular Cardiology	26	5.000	Nature Communications	1,459	14.919	Science	1784	47.728
5	Biophysical Journal	26	4.033	Cell Calcium	1,327	6.817	Cell	1,605	41.584
6	International Journal of Molecular Sciences	25	5.924	Journal of Biological Chemistry	1,115	5.517	Cell Calcium	1,598	6.817
7	Proceedings of the National Academy of Sciences of the United States of America	23	11.205	Cell Metabolism	972	27.287	Cell Reports	1,228	9.423
8	Circulation Research	23	17.367	Molecular Cell	970	17.970	Circulation Research	1,189	17.367
9	Scientific Reports	22	4.380	Journal of Molecular and Cellular Cardiology	934	5.000	PLoS one	1,184	3.240
10	Biochemical and Biophysical Research Communications	19	3.575	PLoS one	790	3.240	EMBO Journal	1,141	11.598

Regarding cocited sources, 120 of 3,557 journals demonstrated over 100 citations, and 30 of them had a citation number over 500. Among them, *Journal of Biological Chemistry* had the highest number of cocitations (*n* = 4,183), Nature (*n* = 3,984) and *Proceedings of the National Academy of Sciences of the United States of America* (PNAS) (*n* = 3,025) occupied the second and third places of journals with the greatest number of citations (Table 4).

### 3.4 Analysis of cocited references

Among 40,354 references, 13 were cited over 100 times. Table 5 shows the top 10 cocited references. The most cocited reference was published in Nature by D. De Stefani (*n* = 230) (De Stefani et al., 2011). All 10 top cocited articles were research articles.

**TABLE 5:** Top 10 co-cited references related to MCU.

Rank	Title	First author	Journal	Cocitation number	Citation number
1	A 40-kDa protein of the inner membrane is the mitochondrial calcium uniporter	Diego De Stefani	Nature	230	1783
2	Integrative genomics identifies MCU as an essential component of the mitochondrial calcium uniporter	Joshua M Baughman	Nature	227	1754
3	EMRE is an essential component of the mitochondrial calcium uniporter complex	Yasemin Sancak	Science	149	593
4	MICU1 and MICU2 finely tune the mitochondrial Ca2+ uniporter by exerting opposite effects on MCU activity	Maria Patron	Molecular Cell	144	465
5	The physiological role of mitochondrial calcium revealed by mice lacking the mitochondrial calcium uniporter	Xin Pan	Nature Cell Biology	137	608
6	MICU2, a paralog of MICU1, resides within the mitochondrial uniporter complex to regulate calcium handling	Molly Plovanich	PLoS One	131	419
7	The mitochondrial calcium uniporter is a multimer that can include a dominant-negative pore-forming subunit	Anna Raffaello	EMBO Journal	128	435
8	MICU1 is an essential gatekeeper for MCU-mediated mitochondrial Ca (2+) uptake that regulates cell survival	Karthik Mallilankaraman	Cel	127	519
9	MICU1 controls both the threshold and cooperative activation of the mitochondrial Ca^2^⁺ uniporter	Gyorgy Csordas	Cell Metabolism	120	441
10	MICU1 encodes a mitochondrial EF hand protein required for Ca (2+) uptake	Fabiana Perocchi	Nature	105	852

**FIGURE 4:**
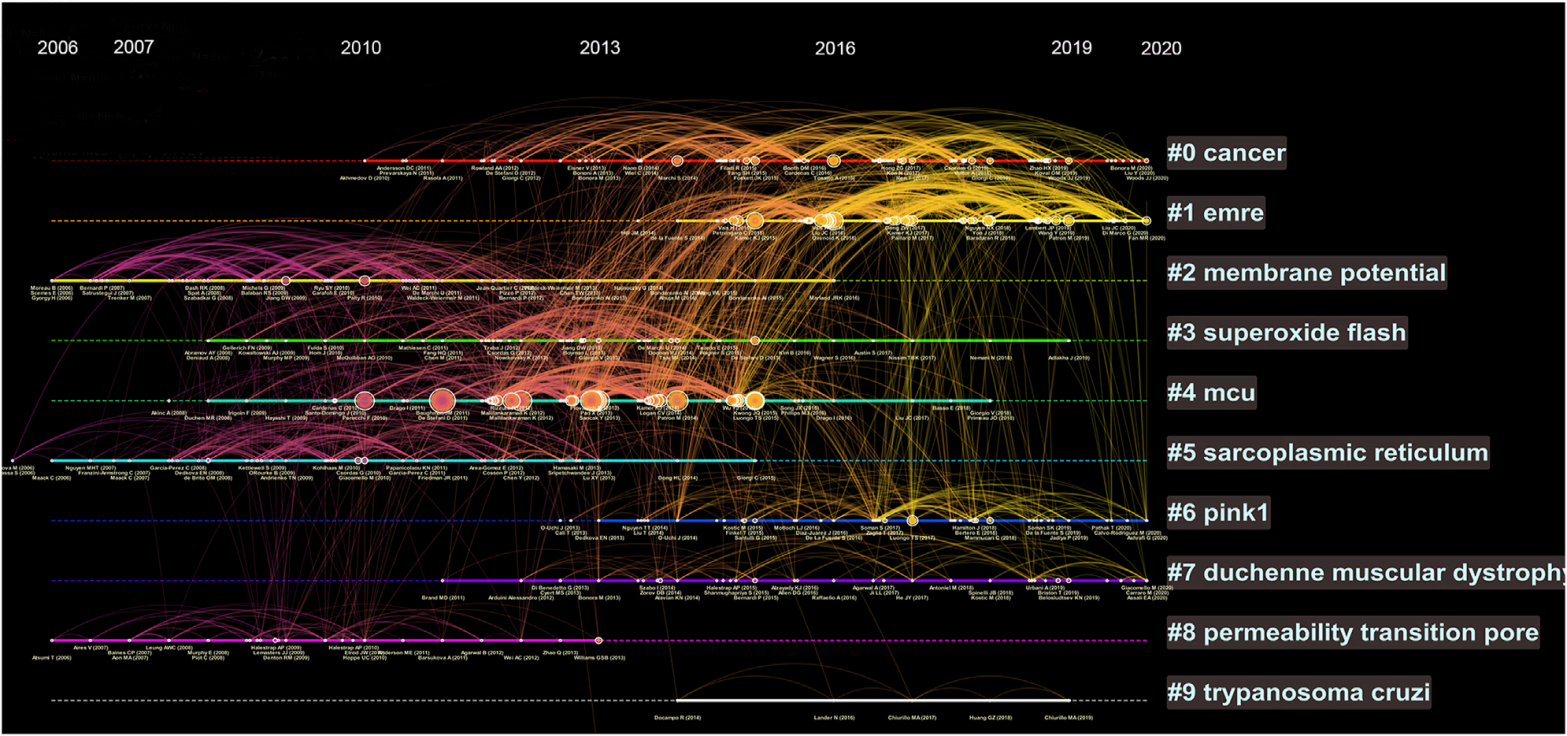
Timeline view of co-cited references. Each horizontal line represents a cluster; the larger the cluster, and #0 is the largest cluster. The node size reflects the co-cited frequencies, and the links indicate the co-cited relationships; the color of node and line represent different years; nodes are at their first co-cited year. Solid line means they are hot clusters in those years.

We further analysed the references by timeline and visualized the relative hotspots by cluster mapping. As shown in Figure 4, the tags of each cluster are the terms with the highest frequency of research. Clusters #0 (cancer) and #1 (EMRE) were the clusters with the highest frequency and are still ongoing, which means they are hotspots and frontiers in current research. In addition, #6 (PINK1) and #7 (Duchenne muscular dystrophy) were notable research topics. The detail of Figure 4 can be seen in Supplementary Figure S1.

**FIGURE 5:**
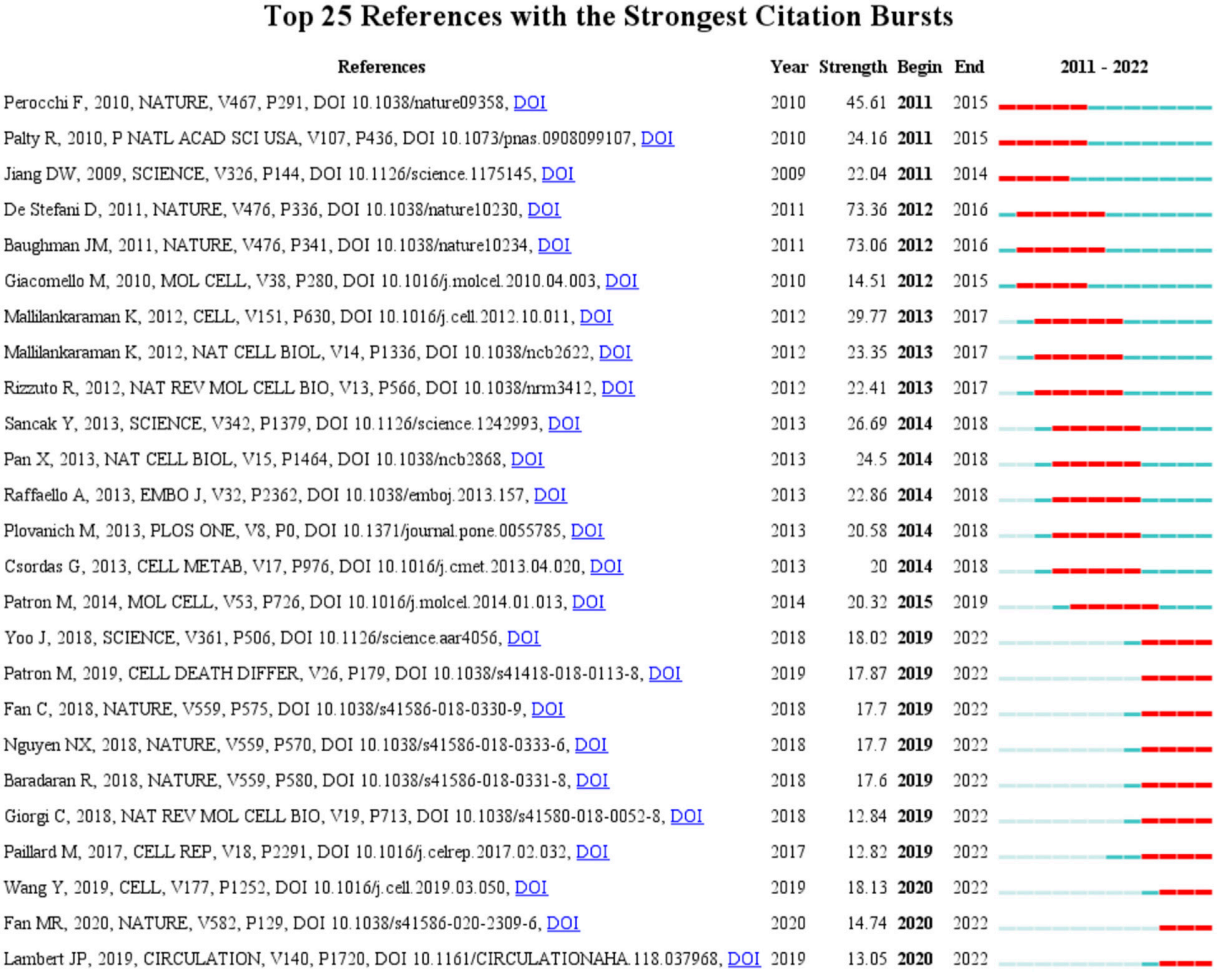
Top 25 references and their strength with the strongest bursts which was in burstness for at least 2 years (sorted by starting year). Red bar displays the years of their burst.

A citation burst indicates that a publication was significantly cited in a specific period (Chen, 2017). Here, we discovered the publication that was significantly cited for over 2 years (Figure 5). The article with the highest citation burst strength was by D. De Stefani and was entitled “A 40-kDa protein of the inner membrane is the mitochondrial calcium uniporter” (strength = 73.36), followed by another article from Nature entitled “Integrative genomics identifies MCU as an essential component of the mitochondrial calcium uniporter” (strength = 73.06) (Baughman et al., 2011). These two articles first reported on the structure and status of MCU, with a citation burst from 2012 to 2016. In the top 25 citation bursts, 10 of them are still in the burst phase. The study currently in a citation burst with the highest strength is an article published in Cell entitled “Structural Mechanism of EMRE-Dependent Gating of the Human Mitochondrial Calcium Uniporter,” which discusses the regulating role of EMRE in MCU (strength = 18.13) (Wang et al., 2019); the article with the second highest strength was published in Science and demonstrated the structure of MCU by cryo-electron microscopy (strength = 18.02) (Yoo et al., 2018).

### 3.5 Analysis of keywords

**FIGURE 6:**
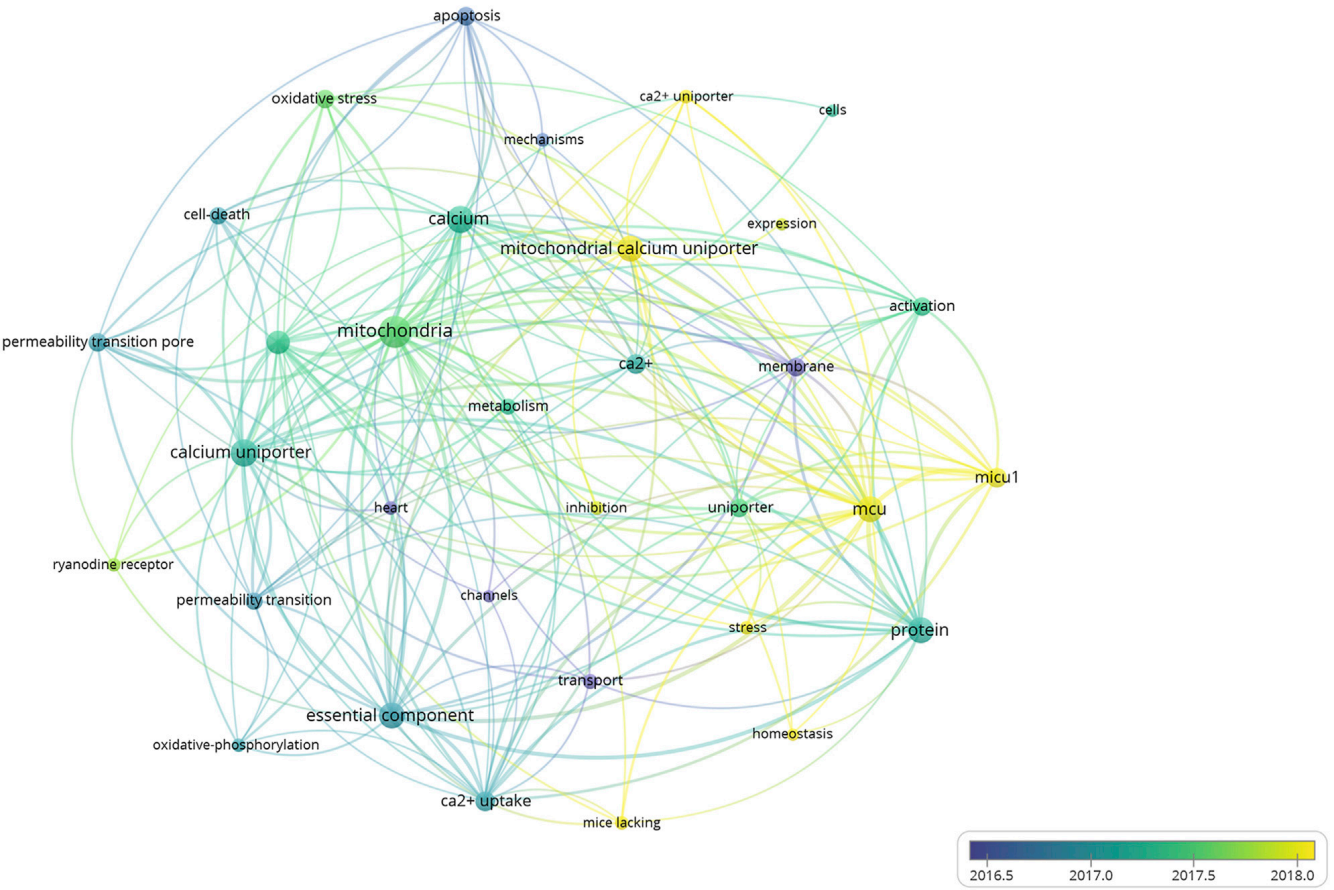
The overlay map of keywords related to MCU. Keywords appeared ≥50, max lines = 200. The node size reflects the co-occurrence frequencies, the thickness of the link reflects co-occurrence frequency, and the color indicates the average published year.

**FIGURE 7:**
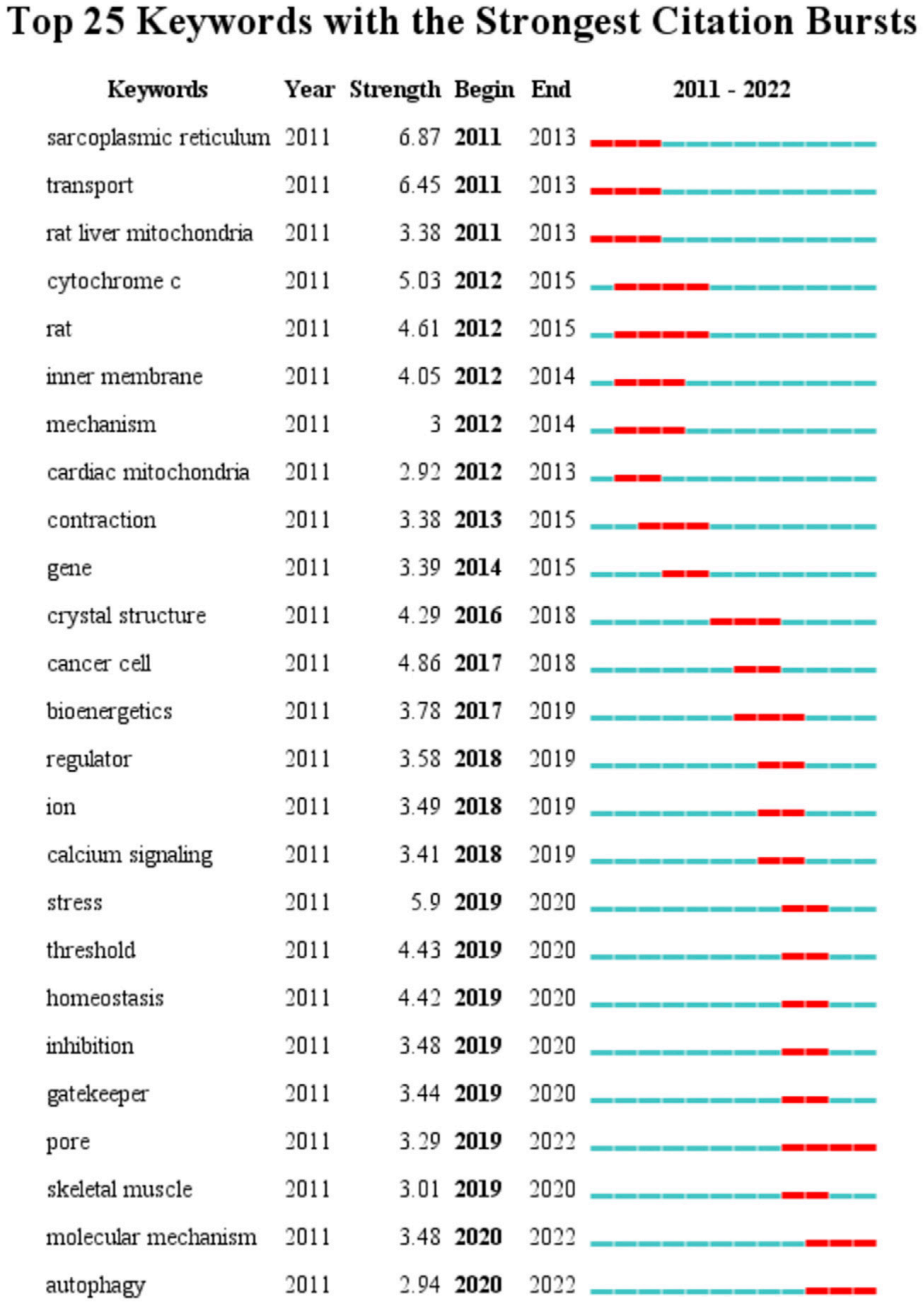
Top 25 keywords and their strength with the strongest bursts which was in burstness for at least 2 years (sorted by starting year). Red bar displays the years of their burst.

A total of 4,151 keywords were distinguished, among which 181 appeared at least 10 times, and 27 keywords appeared 50 times or more. Table 6 shows top 20 keywords related to MCU. Among these 4,151 keywords, endoplasmic reticulum had the most appearances (*n* = 196), followed by permeability transition pore (*n* = 108) and oxidative stress (*n* = 106). Figure 6 shows the keyword overlap map, and the colour indicates the average year of the corresponding keyword appearance. From the figure, we can see that mitochondrial calcium uniporter, MICU1, and homeostasis are yellow-coloured, which means that they are highly focused keywords in recent years. The mechanism of MCU complex and the component on mitochondrial homeostasis attracts interest from researchers these years. In addition, ryanodine receptor and oxidative stress are also two keywords appear recently, indicating the calcium of sarcoplasmic reticulum and mitochondrial metabolism are also two hotspots these years. Figure 7 Shows top 25 keyword with citation burst and the burst period. Keyword bursts are those that were cited significantly frequently over a specific period (Chen, 2017). Over the past 10 years, sarcoplasmic reticulum had the strongest burst (strength = 6.87), followed by transport (strength = 6.45) and stress (strength = 5.9). Notably, pore, molecular mechanism, and autophagy represent current keyword bursts, which indicates that the number of studies on MCU that focused on the regulating mechanism and cell longevity is increasing rapidly these years.

**TABLE 6:** Top 20 keywords related to MCU.

Rank	Keywords	Count
1	endoplasmic reticulum	196
2	permeability transition pore	108
3	oxidative stress	106
4	cell death	105
5	MICU1	103
6	calcium	88
7	permeability transition	82
8	apoptosis	73
9	metabolism	70
10	oxidative phosphorylation	67
11	ryanodine receptor	67
12	homeostasis	46
13	heart	41
14	dynamics	40
15	sarcoplasmic reticulum	39
16	heart failure	38
17	heart mitochondria	38
18	inner membrane	37
19	ATP synthase	36
20	phosphorylation	36

In addition, we summarized pathological processes and molecules regarding MCU studies (Table 7). Regarding pathological processes, oxidative stress (*n* = 106), cell death (*n* = 105), permeability transition (*n* = 82), apoptosis (*n* = 73), and metabolism (*n* = 70) were the 5 keywords with the highest frequencies, suggesting that cellular longevity and mitochondrial homeostasis are research emphasis in the past 10 years. In terms of molecules, MICU1 (*n* = 103), calcium (*n* = 88), ryanodine receptor (*n* = 67), ATP synthase (*n* = 36) and cyclophilin D (*n* = 33) were the top 5 molecules keywords. Among the 5 keywords, MICU1, calcium and ryanodine receptor are highly related to the regulation of mitochondrial calcium homeostasis, cyclophilin D is also associated with permeability transition, which also affects mitochondrial calcium homeostasis (Baines et al., 2005). The results suggest that mitochondrial calcium homeostasis and energy metabolism are hotspots in MCU studies. Moreover, we also analysed top 10 keywords of pharmacological modulation molecules of MCU (Table 8). Over last 10 years, ryanodine receptor (*n* = 67), ATP synthase (*n* = 36) and cyclophilin D (*n* = 33), cyclosporine A (*n* = 29) and inositol 1,4,5-trisphosphate receptor (IP3R) (*n* = 25) were the top 5 keywords. Among the keywords, cyclophilin D and cyclosporine A are associated with the function of mPTP, whereas ryanodine receptor and IP3R are key pharmacological modulation molecules in calcium flux of sarcoplasmic reticulum and endoplasmic reticulum.

**TABLE 7:** Top 10 pathological processes and molecules related to MCU.

Rank	Pathological processes	Count	Molecules	Count
1	oxidative stress	106	MICU1	103
2	cell death	105	calcium	88
3	permeability transition	82	ryanodine receptor	67
4	apoptosis	73	ATP synthase	36
5	metabolism	70	cyclophilin D	33
6	oxidative phosphorylation	67	dependent anion channel	33
7	dynamics	40	cyclosporine A	29
8	phosphorylation	36	inositol 1,4,5-trisphosphate receptor	25
9	calcium signaling	25	reactive oxygen species	19
10	calcium homeostasis	19	ruthenium red	19

**TABLE 8:** Top 10 keywords of pharmacological modulation molecules of MCU.

Rank	Keywords	Count
1	ryanodine receptor	67
2	ATP synthase	36
3	cyclophilin D	33
4	cyclosporine A	29
5	inositol 1,4,5-trisphosphate receptor	25
6	ruthenium red	19
7	mitofusin 2	16
8	Na+/Ca2+ exchanger	12
9	cytochrome C	12
10	complex I	10

## 4 Discussion

### 4.1 General information

From our bibliometric analysis based on the WOSCC, we found that 1,030 articles were published by 5,050 authors from 1,145 affiliations and 62 countries or regions.

Since 2011, studies related to MCU have increased rapidly. As a new established area of research, MCU is gaining increasing interest. The number of articles published in 2021 was almost ten times that published in 2011, and more mechanisms and diseases were studied. It can be expected that more studies related to MCU will be conducted and that this research area will gain public attention.

In our analysis of countries/regions and institutions, we mainly focused on publication number and centrality. The United States contributed most of the MCU studies and published more than twice as many articles as China, which contributed the second most publications. In addition, the centrality of the United States was 0.71, which means that the United States has the most important “bridge” effect in the world network. However, in contrast, the centrality of China was only 0.02 (<0.1) despite a large number of published articles, suggesting that China does not represent a “bridge” effect in the world (Liu et al., 2022). Of the top 10 institutions with the most publications, six were in the United States, three were in Italy and one was in Israel. Of note, the University of Padua had the highest centrality (centrality = 0.2).

In the analysis of authors and cocited authors, Rosario Rizzuto published the most articles and was also the most cocited author. His studies on MCU began in 2011, when he first published the discovery of MCU in Nature. Since then, he has studied the association between mitochondrial calcium uptake and cell responses in various cells, such as cardiomyocytes and pancreatic *β* cells (Drago et al., 2012; Tarasov et al., 2012; Tarasov et al., 2013). In 2014, he published an article in Molecular Cell, which revealed the function of MICU1 and MICU2 and explained their role in calcium regulation in mitochondria (Patron et al., 2014). This article was No. 4 in the list of most cited articles. In 2016, Rosario Rizzuto and his group published a review entitled “Enjoy the Trip: Calcium in Mitochondria Back and Forth,” which summarized the regulatory mechanism of calcium homeostasis and has been cited 80 times (De Stefani et al., 2016). Regarding articles published recently, Wang et al. published an article in 2019 that showed the cryo-EM structure of the MCU-EMRE complex, which has had an impact since 2019 (Wang et al., 2019). This article also had the strongest burst among articles with a citation burst up to 2022.

In the analysis of journals, Cell Calcium published most of the articles related to MCU and was ranked No. 5 in the list of most cited journals. Nature and *PNAS* were both in the top five cited journals and cocited journals. The number of citations in Nature was more than twice that in *PNAS*, which reveals that the two publications that demonstrated the existence and important role of MCU promoted great interest in this category (Baughman et al., 2011; De Stefani et al., 2011).

In keyword analysis, endoplasmic reticulum had the most appearances, followed by permeability transition pore and oxidative stress. Since MCU is a newly found molecule, the regulation mechanism of mitochondrial calcium homeostasis is the topic of most interest. The components and regulating factors of MCU have attracted the attention of researchers in the last decade. In terms of keyword bursts, sarcoplasmic reticulum had the highest strength. The sarcoplasmic reticulum and mitochondria play important roles in calcium flow in cells. It has been found that mitochondria can buffer sarcoplasmic reticulum-derived diastolic calcium release to mitigate calcium-dependent pathological remodeling, which is mediated by mPTP-depended calcium efflux in polymorphic ventricular tachycardia mice (Tow et al., 2022). Notably, autophagy represents a keyword that is currently in a keyword burst. Autophagy is a vital process in maintaining cell organism homeostasis and quality, and MCU has been proven to be a regulating factor of autophagy (Gherardi et al., 2019). This finding indicates that the role of MCU in autophagy is worth more attention and further exploration. Regarding cellular processes, the association with MCU and mitochondrial function is highly concentrated. In addition, cellular longevity is also focused. With respect to molecules and pharmacological modulation, the role of MCU in calcium flux attracts interest over last 10 years. In particular, the association of MCU and mPTP and the calcium flux of sarcoplasmic reticulum and endoplasmic reticulum are hotspots of mechanism studies.

### 4.2 Current studies and research trends

Mitochondria take part in various cellular processes, such as ATP production, apoptosis, and necrosis (Nunnari and Suomalainen, 2012). In particular, mitochondrial calcium plays a key role in the regulatory function of mitochondria (Zhang et al., 2022b). Calcium shuttles between the cytoplasm and mitochondria to maintain a steady level and regulates cellular activities. The concentration of calcium affects aspects such as mitochondrial membrane permeability and ATP production (Sheu and Jou, 1994; Jouaville et al., 1999). Thus, homeostasis of calcium in mitochondria is necessary for normal cellular metabolism, which is modulated by calcium uptake and efflux.

According to our analysis, cancer is currently one of the research hotspots and was the most heated cluster. In cancer cells, MCU expression is associated with proliferation, metastasis, migration, ROS production, etc. Downregulation of MCU has been proven to be effective in inhibiting the proliferation and migration of SKOV3 ovarian cancer (Wang et al., 2022a). Wang et al. demonstrated that MCU expression leads to accelerated invasion and metastasis and decreased oxidative stress in pancreatic ductal adenocarcinoma (PDAC) cells (Wang et al., 2022b). MCU expression is also related to overexpression of vascular endothelial growth factor (VEGF) and matrix metalloproteases 2 (MMP2), which leads to angiogenesis and invasion of oesophageal cancer (Liu et al., 2020; Miao et al., 2021). In colorectal cancer cells, MCU expression is also related to poor prognosis. Liu et al. showed that MCU is upregulated in colorectal cancer cells, which promotes mitochondrial biogenesis and further facilitates tumour growth (Liu et al., 2020). MCU was also found to be associated with hypoxia-inducible factor-1α (HIF-1α) expression, which enhances tumour growth and lymph node infiltration of triple-negative breast cancer (Tosatto et al., 2016). In contrast, some drugs have been shown to have anticancer effects by upregulating the expression of MCU. In MDA-MB-231 cells (breast cancer cells) treated with RY10-4, MCU expression is enhanced, which leads to calcium overload, ROS accumulation, loss of mitochondrial transmembrane potential (mTP) and excessive opening of the mPTP. The aforementioned cell responses contribute to cell apoptosis (Xue et al., 2021). Similar results were also found in *Tetrastigma hemsleyanum-*treated HepG2 cells (Li et al., 2021b).

PINK1 (PTEN-induced putative kinase 1) represented another hotspot cluster in our analysis. PINK1 is a key regulator in mitophagy and plays a crucial role in the quality control of mitochondria (Ge et al., 2020; Terešak et al., 2022). The PINK1/parkin pathway is involved in the elimination of dysfunctional mitochondria, which has been proven to be a key mechanism in the development of Parkinson’s disease (Eldeeb et al., 2022). In PINK1 and parkin knockout mutants, features related to apoptosis, such as increased apoptosis and increased sensitivity to mitochondrial damage, have been detected (Park et al., 2006). Soman et al. reported that inactivation of MCU leads to the prevention of dopaminergic neuronal cell loss in PINK^−/-^ zebrafish and that the function of the mitochondrial respiration chain was also improved (Soman et al., 2017). Degeneration of dopaminergic neurons is reduced by nicotine, the mechanism of which is upregulation of MCU and PINK1. The activation of PINK1 further decreases mitochondrial stress and acts as a protector in mitochondria (Nourse et al., 2021). Another element associated with neurotoxicity, lanthanum (La), leads to the impairment of learning and memory. The potential mechanism may be attributed to overexpression of MCU and overactivation of the PINK1/parkin pathway, causing excessive mitophagy (Yu et al., 2020). Moreover, the MCU and PINK1/parkin pathways are involved in heart failure. MCU has been reported to be upregulated in heart failure rats, and the application of Ru360 (MCU inhibitor) alleviates the damaged cardiac function. Cardiac protection may be related to the upregulation of PINK1 and parkin, which promotes mitochondrial integrity (Yu et al., 2018). In summary, themes on the MCU complex and PINK1 are potential hotspots owing to their participation in various diseases and complicated mitochondrial regulation mechanisms. Neurodegeneration diseases and cardiovascular diseases are attracting interest from researchers and might be a trending topic.

The role of MCU in cardiovascular diseases also arouses interest over last 10 years. Calcium overload is one of the key pharmacological processes in ischemia-reperfusion injury (I/R injury) (Wang and Zhou, 2020). It has been found that the suppression of MCU prevented the abnormal opening of mPTP and protected microcirculation in cardiac microvascular endothelial cells (CMECs) treated with I/R (Li et al., 2020). Rasmussen et al. also demonstrated that in I/R-treated dominant negative-MCU mice, inner mitochondrial membrane potential was preserved and ROS production was reduced (Rasmussen et al., 2015). Similarly, displacement of MCU by MCUb prevented calcium overload and decreased infarct size after ischemia/reperfusion injury. However, mitochondrial energetics and contractile function were induced by acute decreases in calcium uptake, which indicated that the displacement might be a stress-responsive mechanism (Lambert et al., 2019). In addition, MCU has been proven as an essential component in generation and maintenance of heart rhythm. MCU enhances oxidative phosphorylation and accelerates reloading of an intracellular calcium compartment, which is required for heartbeat (Wu et al., 2015). Accordingly, abnormal rhythm resembling episodes of sinus arrest was detected in MCU-KO zebrafish, with damaged myofibrils and swollen mitochondria presented (Langenbacher et al., 2020). Thus, as an essential component in modulation of mitochondrial calcium homeostasis, MCU may be a promising research target in physiological and pathological processes in cardiovascular diseases.

### 4.3 Strengths and limitations

To our knowledge, this is the first bibliometric analysis of studies related to MCU. We analysed the trends and hotspots related to this area of research. In addition, we visualized the current study topic and keywords by using bibliometric software and provided results in multiple dimensions. Compared to traditional reviews, our study provides an overview of MCU research, which serves as a comprehensive and objective summary of current studies. Our analysis may also contribute to further development in this field of research.

However, our study has limitations. We only analysed publications in English from the WOSCC, which may give rise to the omission of articles in languages other than English and not collected in the WOSCC. Non-etheless, since most of the articles are written in English, the WOSCC is the most common source of publications in bibliometric analyses, and the results of our analysis have representativeness to a large extent (Ke et al., 2020).

## 5 Conclusion

In conclusion, in the past decade, the number of MCU studies has been increasing steadily, and cooperation worldwide is also active. The United States has contributed the most published articles and has the highest centrality. Rosario Rizzuto not only has published most of the articles but also is the most cocited author. Current studies focus on the structure of the MCU complex and its regulatory effect on mitochondrial calcium homeostasis. In addition, the potential role of MCU in different diseases is being explored. Current research hotspots are associated with cancer and neurodegenerative diseases; however, studies on cardiovascular diseases are also attracting interest. Our analysis provides guidance and new perspectives for further research on MCU.

## Data Availability

The raw data supporting the conclusion of this article will be made available by the authors, without undue reservation.
