# Supplementary figures and images for: Knowledge mapping of mitochondrial calcium uniporter from 2011 to 2022: A bibliometric analysis
Source: Front Physiol. 2023 Jan 20;14:1107328. doi: 10.3389/fphys.2023.1107328 (PMC9895098; doi:10.3389/fphys.2023.1107328)

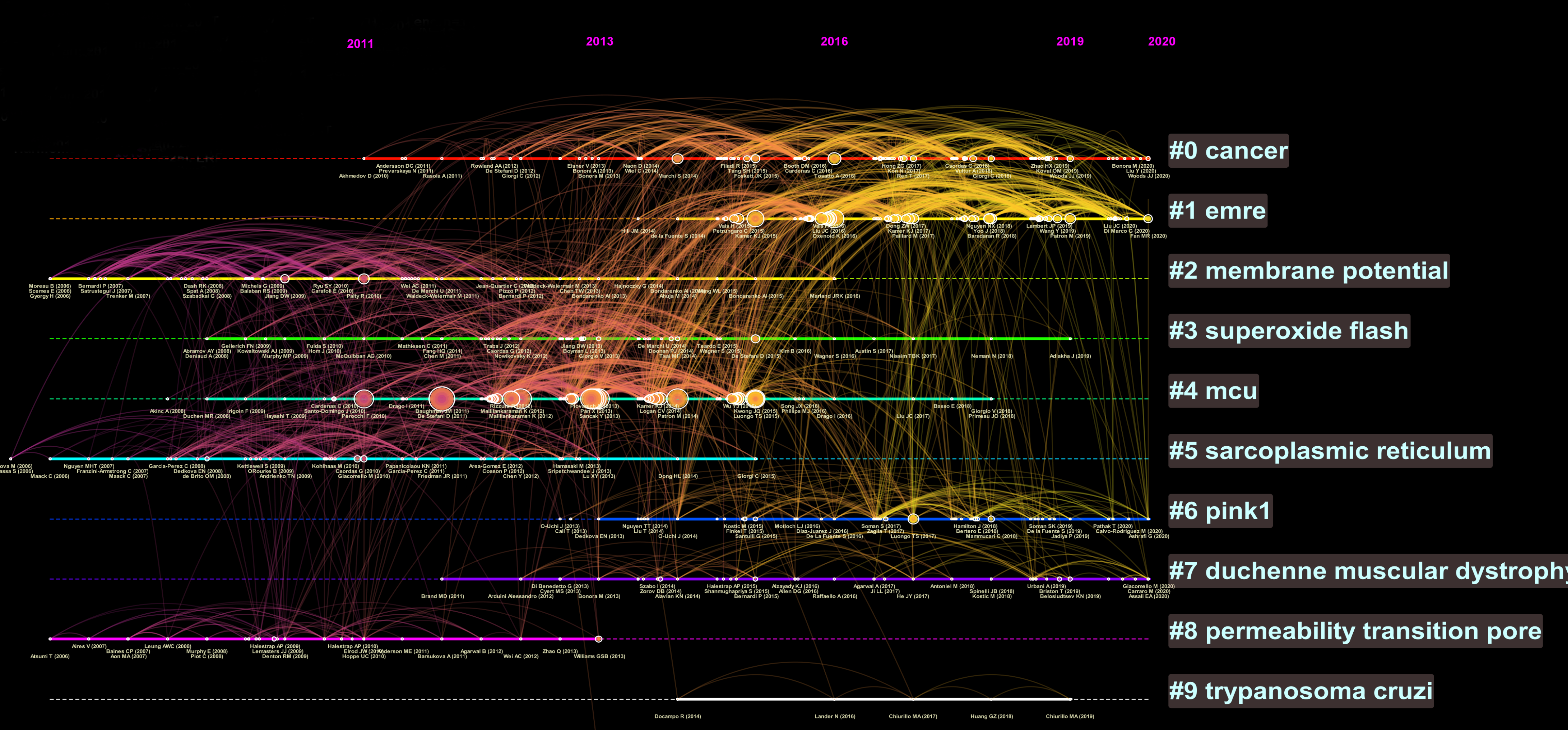

Supplement: Supplementary file 1 [file Image1.PNG]
